# Gut microbial composition in patients with psoriasis

**DOI:** 10.1038/s41598-018-22125-y

**Published:** 2018-02-28

**Authors:** Francisco M. Codoñer, Ana Ramírez-Bosca, Eric Climent, Miguel Carrión-Gutierrez, Mariano Guerrero, Jose Manuel Pérez-Orquín, José Horga de la Parte, Salvador Genovés, Daniel Ramón, Vicente Navarro-López, Empar Chenoll

**Affiliations:** 1 Lifesequencing S.L.-ADM, Catedrático Agustín Escardino Benlloch 9 Edif 2, 46980 Paterna, Valencia Spain; 2 Department of Clinical Medicine, MiBioPath group, Universidad Católica San Antonio de Murcia (UCAM), Guadalupe, Spain; 3 Department of Dermatology, Hospital Universitario del Vinalopó, Elche, Spain; 4 Especialidades Farmacéuticas Centrum, Alicante, Spain; 5 Korott S.L., Carrer Filà Benimerines, 61, 03801 Alcoi, Alicante Spain; 6 Department of Pharmacology, Pediatrics and Organic Chemistry, Universidad Miguel Hernández de Elche, Alicante, Spain; 7 Biopolis S.L.-ADM, Catedrático Agustín Escardino Benlloch 9 Edif 2, 46980 Paterna, Valencia Spain; 8 Infectious Disease Unit, Hospital Universitario del Vinalopó, Elche, Spain

## Abstract

Since the last 5–10 years the relevance of the gut microbiome on different intestinal illnesses has been revealed. Recent findings indicate the effect of gut microbiome on certain dermatological diseases such as atopic dermatitis. However, data on other skin diseases such as psoriasis are limited. This is the first time attempting to reveal the gut microbiome composition of psoriatic patients with a prospective study including a group of patients with plaque psoriasis, analyzing their gut microbiome and the relationship between the microbiome composition and bacterial translocation. The microbiome of a cohort of 52 psoriatic patients (PASI score ≥6) was obtained by 16s rRNA massive sequencing with MiSeq platform (Illumina inc, San Diego) with an average of 85,000 sequences per sample. The study of the gut microbiome and enterotype shows from the first time a specific “psoriatic core intestinal microbiome” that clearly differs from the one present in healthy population. In addition, those psoriatic patients classified as belonging to enterotype 2 tended to experience more frequent bacterial translocation and higher inflammatory status (71%) than patients with other enterotypes (16% for enterotype 1; and 21% for enterotype 3).

## Introduction

Psoriasis is considered a systemic autoimmune inflammatory and proliferative chronic disease^1–3^. Although the pathogenesis of psoriasis is not well understood, it is hypothesized to be multi-factorial, influenced by genetic and immunological factors. This condition is characterized by epidermal and dermal infiltrations of activated T lymphocytes^4,5^. Bacterial DNA translocation (BT) in blood samples has recently been described in patients with psoriasis^6^. The results of this work suggest that new outbreaks of active plaque psoriasis may be related to the presence of circulating bacterial DNA in blood, originated from the intestinal lumen^6^. This indicates the key role played by healthy intestinal bacterial composition in reducing intestinal permeability and the risk of BT.

In recent years, in-depth studies have assessed the association between the gut microbiome and certain disorders such as cirrhosis^7^, obesity^8^, inflammatory bowel diseases^9^, diabetes^10^ and central neuronal disorders^11^ among others. Recently, a study focused on autism and accompanying GI symptoms^12^, found that autism patients had a distinct and less diverse gut microbial composition with lower levels of *Prevotella*, *Coprococcus*, and an unclassified *Veillonellaceae*. All these results suggest a potentially important role of the gut microbiome in illnesses that are not directly related to the digestive tract.

This work attempts to describe the intestinal microbial composition of psoriatic patients and the putative relation between the BT and disease worsening.

## Results

A total of 52 patients previously diagnosed with plaque psoriasis by clinical, laboratory and/or histological findings were included (Supplementary Table S1). Bacterial DNA was detected in 13 blood samples (13/52 = 25%) being considered as BT-positive patients. The rest were considered as BT-negative group.

**Figure 1:**
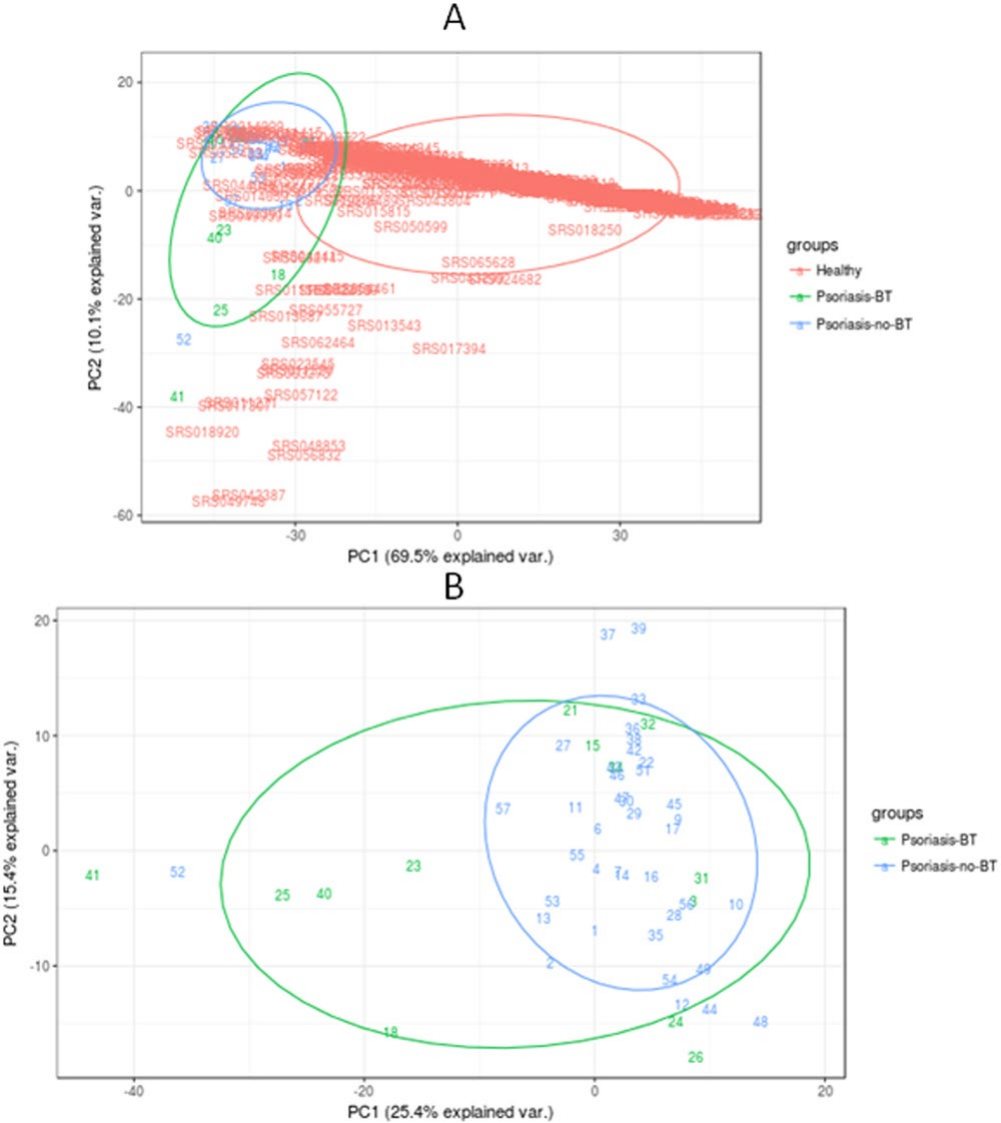
Principal Component Analysis of the microbiome composition comparing (**A**) The variability of the intestinal bacteria composition of healthy population (red) (extracted from the Human Microbiome Project) compared with patients that undergone BT (in green) and with those that do not have bacterial translocation (in blue). This statistical analysis was performed using R package version 3.2.3, comparing the bacteria composition at the genera taxonomical level for each patient in order to look for groups of genera that can allow grouping samples based on the variability found. PC1, or Principal Component 1, explained the 69.5% of the total variability found, and PC2 or Principal Component 2, explained the 10.1% of the total variability. (**B**) The variability of the intestinal bacteria composition in patients that undergone BT (in green), compared with those that do not have bacterial translocation (in blue). This statistical analysis was performed in R package version 3.2.3, comparing the bacteria composition at the genera taxonomical level for each patient in order to look for groups of genera that can allow groping samples based on the variability found. PC1, or Principal Component 1, explained the 25.4% of the total variability found, and PC2 or Principal Component 2, explained the 15.4% of the total variability.

Firstly, the intestinal bacterial composition in psoriatic patients was analyzed by massive genome sequencing based on 16s rRNA, inspecting a mean of over 85,000 sequences per sample (raw and clean number of sequences, mean length, total mega bases sequenced and mean quality per sample can be found at Supplementary Table S2 and S3 respectively) and comparing based on BT detection (Fig. 1A). The comparison did not reveal differentiated BT-positive and BT-negative populations; the latter population being nested within the former. This allowed us to describe a “psoriatic core microbiome” at genus level based on mean values of the bacteria detected in each patient as well as with the median of all patients (Supplementary Table S4). The bacterial composition of all psoriatic patients was then compared with a cohort of over 300 healthy individuals extracted from the human microbiome project (http://hmpdacc.org/). In this case, differences were observed in the intestinal bacterial composition between healthy (as considered at the Human Microbiome Project) and psoriatic groups, although some healthy people clustered closer to psoriatic patients (Fig. 1B). In general, focusing on the PCA analysis, in psoriatic patients we detected a decrease in the genus *Bacteroides* and an increase in *Akkermansia* spp. compared with the healthy group. Shannon biodiversity index was calculated (included in Supplementary Table S5) and the comparison (median, (IQ)) between healthy people and psoriatic patients rendered a significantly higher variability (p-value < 0.001) in psoriatic patients (2.94, (2.75–3.14)) compared with the healthy population (1.92, (1.27–2.41)). Moreover, we observed significant differences when comparing the diversity of BT-positive and BT-negative patients (p value = 0.013), where the variability was lower and more stable in BT-positive patients (2.83, (2.47–2.92)) than in BT-negative patients (2.99, (2.78–3.19)).

**Figure 2:**
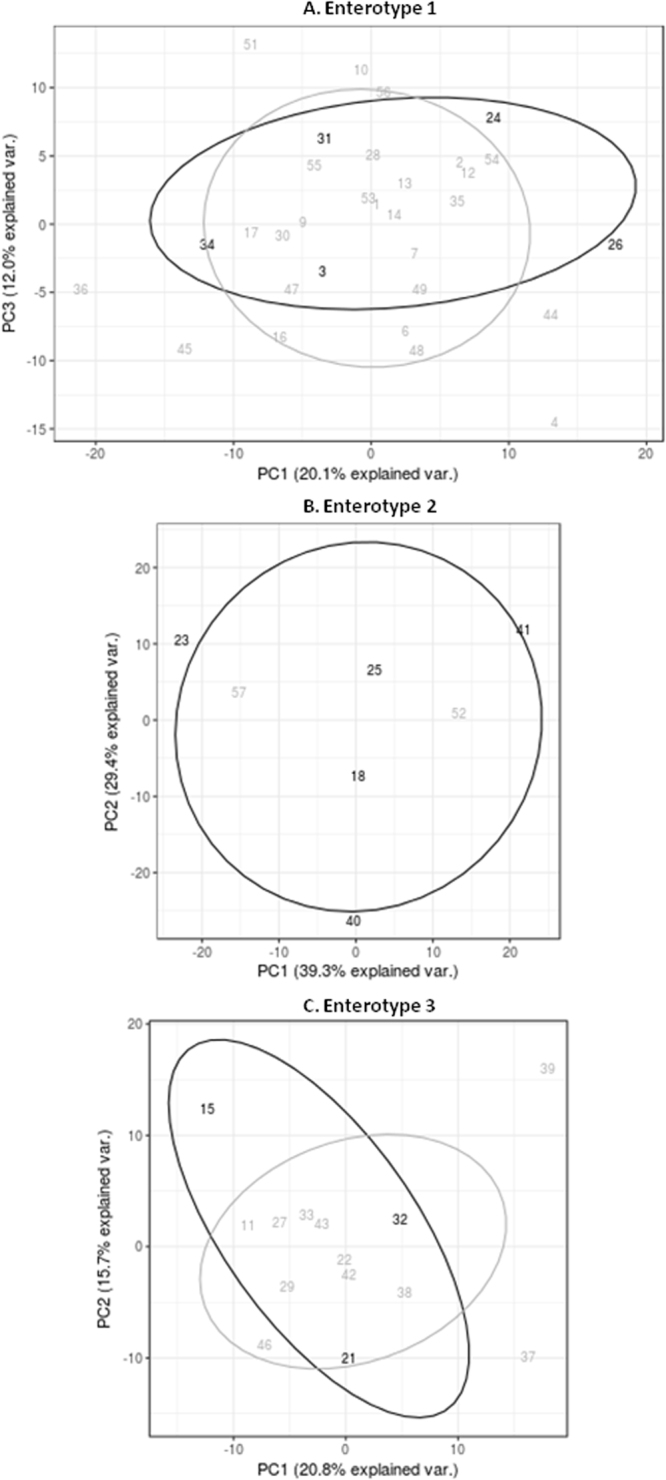
Principal Component Analysis of the microbiome composition comparing the variability of the intestinal bacteria composition of patients that undergone BT (in black) and with those that do not have bacterial translocation (in grey), differentiating between the three enterotypes, Ent1 (**A**), Ent2 (**B**) and Ent3 (**C**). This statistical analysis was performed using R package version 3.2.3, comparing the bacteria composition at the genera taxonomical level for each patient in order to look for groups of genera that can allow grouping samples based on the variability found. When comparing patients belonging to Ent1, PC1, or Principal Component 1, explained the 20.1% of the total variability found, and PC2 or Principal Component 2, explained the 12.0% of the total variability. Taken into account those that are associated to Ent2, PC1, or Principal Component 1, explained the 39.3% of the total variability found, and PC2 or Principal Component 2, explained the 29.4% of the total variability, where as those belonging to Ent3, PC1, or Principal Component 1, explained the 20.8% of the total variability found, and PC2 or Principal Component 2, explained the 15.7% of the total variability.

The intestinal bacterial composition was compared based on BT detection and enterotype classification (Arumugam *et al*.)^13^: enterotype 1 (predominance of *Bacteroides*, Fig. 2A), enterotype 2 (predominance of *Prevotella*) (Fig. 2B) and enterotype 3 (predominance of *Ruminococcus*) (Fig. 2C). In no case, PCA analysis did show a strong separation between between BT+ and BT− psoriatic subjects. Regarding patients grouped in enterotype 2, we found a higher BT presence (71.4% of patients; 5/7) compared with patients classified in the other enterotypes (16.1% (5/31) for enterotype 1; p-value < 0.05 and 21.4% (3/14) for enterotype 3; p-value =< 0.076).

**Figure 3:**
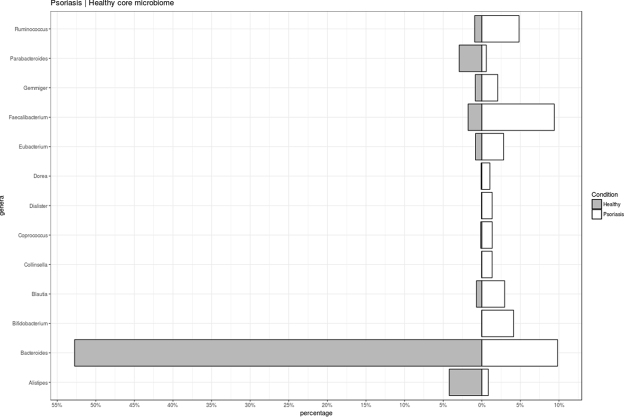
Microbiome characterization, significant genera differentially present in healthy and psoriatic population. Median percentages values of different significant genera are indicated.

Further comparison of psoriatic *versus* healthy microbiome composition was performed (Fig. 3). The analysis showed some genera that were differentially present at psoriatic individuals, being of relevance the increase presence of *Faecalibacterium* and the decrease of *Bacteroides*. In the same sense, we compared those psoriatic patients classified in any of the enterotypes with the healthy individuals classified in the same enterotype (Supplementary Figure S1) and in all cases, psoriatic patients showed an increase presence of *Faecalibacterium* and a decrease of *Bacteroides*. Analyzing differences in bacterial composition between healthy and psoriatic subjects, and among the enterotypes, although in all cases *Faecalibacterium* and *Bacteroides* changes their percentages, the highest ratio between *Faecalibacterium* and *Bacteroides* levels was observed in enterotype 2 psoriatic subjects.

Finally, we compared the microbial composition of BT+ psoriasis subjects versus healthy group, and BT− psoriasis subjects versus healthy groups (Supplementary Files 7 and 8). In the case of BT+ psoriasis subjects, genus *Streptococcus* showed 7.25 log2 Fold Change (−log10(p-value) = 20.35), but low incidence in terms of quantity in core microbiome, followed by *Bifidobacterium* (6.95 log2 Fold Change; −log10(p-value) = 6.60). Genus *Akkermansia* showed 5.28 log2 Fold Change (−log10(p-value) = 3.39). In the case of BT− psoriasis subjects, although *Methanobrevibacter* was the genus with higher differences in log2 Fold Change (10.75 log2 Fold Change, −log10(p-value) = 11.95), its representation was scarce in final psoriatic core microbiome. Although the rest of groups remained very similar in both comparisons, genus *Clostridium* showed 6.12 log2 Fold Change in the case of BT+ versus healthy subjects (−log10(p-value) = 14.36) and 3.62 log2 Fold Change when comparing BT− versus healthy subjects (−log10(p-value) = 15.41). Differences were shown when comparing separately psoriatic BT+ and BT− core microbiome versus healthy group (Supplementary File 9). Psoriatic BT− core microbiome was very similar to the one including both BT+ and BT− subjects. In the case of BT+ versus healthy comparison, *Parabacteroides*, *Collinsella, Blautia* and *Ruminococcus* groups were not differentially present.

## Discussion and Conclusions

In order to evaluate the differences in psoriatic gut microbiome and its relation with BT, the microbiome of a cohort of 52 psoriatic patients has been analyzed in this work. The results of this preliminary analysis show a defined microbial structure in patients with psoriasis, and that this “psoriasis microbiome” clearly differs from the healthy population. Moreover, the relation between this microbial composition and bacterial translocation (BT) from the intestinal lumen is shown.

Compared with healthy data, the psoriatic microbiome was shown more diverse than healthy population. In a study conducted with 15 patients with skin psoriasis, Scher *et al*.^14^ found a decreased in bacterial diversity when compared to that in healthy controls. These differences could be explained by different factors: i) in our study, psoriatic severity was higher (PASI mean 6.3 in Scher *et al*.^14^ vs. 13.3 in our study), and that could imply differences in gut microbiome, and ii) differences in massive sequencing platform and sequenced region (V1-V2 in Scher *et al*.^14^ vs. V3-V4 in our study). The psoriatic microbiome obtained in our study was defined by an increase presence of *Faecalibacterium* and a decrease of *Bacteroides*. The genera *Akkermansia* and *Ruminococcus* showed higher values in psoriatic patients, on the contrary to Scher *et al*.^14^ findings, that found lower levels of both groups in psoriatic and arthritis psoriatic patients. *Faecalibacterium* species has been shown at levels of around 5% from the gut bacterial population^15^ and remarkably its increase in gut microbiota has been associated with immune-regulation. Previous works have analyzed the role of *Faecalibacterium* (*F. prausnitzii*) in inflammation, with a negative correlation between the abundance of this group and irritable bowel syndrome and celiac disease, among others^16^. Other researchers have found that an increase in species belonging to this genus is associated with inflammation diseases such Chron disease^17^. Regarding skin-associated diseases, a dysbiosis in *Faecalibacterium* subspecies have been shown to have enriched percentages in infants with eczema^18^ and an impact on gut epithelial barrier and as a result on atopic dermatitis^19^. In this study, Shong *et al*. found compositional changes in *Faecalibacterium prausnitzii* in atopic dermatitis patients, always with increased levels at the genera level in these patients compared with non-atopic dermatitis patients (following our findings in psoriasis), but with an impact lowering high butyrate and propionate *Faecalibacterium* strain producers. Butyrate and propionate are short chain fatty acids produced by gut microbial and with a demonstrated anti-inflammatory role^19^ and, furthermore, butyrate has shown to be a key-player for maintaining barrier integrity^20^. In this context, a reduction of both butyrate and propionate microbiota producers may have an impact on pro-inflammatory state in gut and consequently a lost in barrier integrity. Regarding *Bacteroides*, Zheng *et al*.^17^ found lower levels of *Bacteroides fragilis* in infants with eczema while two other *Bacteroides* species were enriched pointing to species-specific role and pointed to Th1/Th2 imbalance due to *B. fragilis* levels. In our case, the results point to a relationship between the increase in *Bacteroides* and healthy status.

The comparison of BT+/BT− psoriatic subjects versus healthy population didn’t identify specific bacteria for bacterial translocation. *Parabacteroides*, *Collinsella, Blautia* and *Ruminococcus* groups were differentially present only in BT− subjects but, considering that BT+ represented only 25% of psoriatic people included in the study, these differences must be considered as a punctual result, and further studies should be applied to confirm. Analyzing differences within different enterotypes, our results suggest that psoriatic patients with a relevant presence of *Prevotella* in the intestinal microbiome (enterotype 2) and lower ratio *Bacteroides/Faecalibacterium*, can be more prone to BT.

All these results suggest that bacterial translocation in psoriasis is not induced by one specific microbial group; the event probably is caused by a microbial imbalance among different groups in gut, which may alter organic acid compounds and other molecules, acquiring an inflammation state which leads to bacterial translocation events, among others. Although in our study PASI scores were similar between BT+ and BT− subjects (BT+, PASI average 12.51 ± 4.19; BT−, PASI average 12.77 ± 4.64), it has been previously reported that BT boosts the pro-inflammatory response, promoting skin inflammation, and consequently these patients may require a more aggressive treatment^6^. Therefore, the detection of BT could play a crucial role in a patient’s response to treatment. If we take into account that patients classified in enterotype 2 have a greater likelihood of bacterial DNA being present in blood, an inspection of the bacteria present in stools could be used as one of the follow-up indicators in psoriatic patients, to detect BT events and a stronger inflammatory response.

All in all, the knowledge here communicated, holds conceivable promise for developing novel prognosis and most efficient therapeutics avenues, including antibiotics, probiotics and prebiotics for psoriasis treatment.

## Methods

### Study population

The patients evaluated for inclusion in this study had previously been diagnosed with plaque psoriasis by clinical, laboratory and/or histological findings; they had a PASI score ≥6 and they attended a dermatological clinic from September 2014 to December 2014. A peripheral blood sample was collected from all participants and analyzed for routine biochemical laboratory values, as well as for interleukin (IL) 1β, IL-6, IL-12, IL-23, tumor necrosis factor, and interferon-γ levels. An aliquot of blood was inoculated under aseptic conditions in sterile, rubber-sealed Vacutainer SST II tubes (BD Diagnostics) to detect and identify the origin of bacterial DNA fragments in blood. Finally a stool sample was obtained from all patients and immediately frozen and stored at −80 °C until their processing for massive genome sequencing. Exclusion criteria were the following: the use of immunosuppressant drugs such as systemic corticosteroids, methotrexate, cyclosporine or anti-tumor necrosis factor alpha (TNFα) drugs in the previous 3 months, systemic antibiotics in the previous 2 weeks, and the concomitant diagnosis of cirrhosis, intestinal bowel disease and signs of bacterial infection.

The study was run in accordance with the Helsinki Declaration, as amended in successive world assemblies. The protocol received approval from the Ethics Committee for Clinical Research (CEIC) of the Hospital General Universitario de Alicante, and the “Agencia Española del Medicamento” (Spanish Medicines Agency). All patients gave their written informed consent before their inclusion in the study. There was no change to the trial protocol after it commenced. All methods were performed in accordance with the relevant guidelines and regulations.

### Bacterial DNA identification in peripheral blood

To detect and identify the origin of bacterial DNA fragments in blood, we performed a broad-range polymerase chain reaction and nucleotide sequencing analysis according to the methodology previously described^21,22^. Briefly, DNA was isolated with QIAmp DNA Blood Mini Kit (QIAgen, Hilden, Germany) and a broad-range PCR amplification of the bacterial 16s rRNA gene conserved region was performed using the following primers: 5′-TTCCGGTTGATCCTGCCGGA-3′ as forward, and 5′-GGTTACCTTGTTACGACTT-3′ as reverse. Bacterial DNA fragments were purified with QIAquick purification kit (QIAgen) and purified amplicons were used for the sequencing reactions with Big Dye Terminator v3.1 Cycle Sequencing kit (Applied Biosystems, Foster City, CA, US). The same reverse oligonucleotide for PCR amplification was used as a sequencing primer. The final product was analyzed in the ABI PRISM 310 automated sequencer (Applied Biosystems). Sequences obtained were compared with the database from the National Center for Biotechnology Information (NCBI, www.ncbi.nih.gov) using the advanced BLAST search tool.

### Massive genome sequencing of stool samples of patients with psoriasis

DNA from stool samples was isolated following Yuan and coworkers^23^ with minor modifications, with the aid of MagnaPure Compact System (Roche Life Science), to avoid bias in DNA purification toward misrepresentation of gram-positive bacteria. For massive sequencing, the hypervariable region V3-V4 of the bacterial 16s rRNA gene was amplified using key-tagged eubacterial primers^24^ and sequenced with a MiSeq Illumina Platform, following the Illumina recommendations for Library preparation and sequencing for metagenomics studies. The bacterial composition of all patients was compared with a cohort of healthy sex- and age-matched individuals extracted from the human microbiome project (http://hmpdacc.org/).

### Bioinformatics

The resulting sequences were split taking into account the barcode introduced during the PCR reaction, while R1 and R2 reads were overlapped using PEAR program version 0.9.1^25^ providing a single FASTQ file for each of the samples. Quality control of the sequences was performed in different steps, (i) quality filtering (with a minimum threshold of Q20) was performed using fastx tool kit version 0.013, (ii) primer (16s rRNA primers) trimming and length selection (reads over 300 nts) was done with cutadapt version 1.4.1^26^. These FASTQ files were converted to FASTA files and UCHIME program version 7.0.1001 was used in order to remove chimeras that could arise during the amplification and sequencing step. Those clean FASTA files were BLAST^27^ against NCBI 16s rRNA database using blastn version 2.2.29+. The resulting XML files were processed using a python script developed by Lifesequencing S.L.-ADM (Paterna, Valencia, Spain) in order to annotate each sequence at different phylogenetic levels (Phylum, Family, Genus and Species).

### Statistical Analysis

Descriptive quantitative variables were expressed as means ± SD and as frequencies for qualitative variables. Comparisons between groups were performed with U Mann-Whitney test for quantitative data, while qualitative variables were analyzed using the chi-square and Fisher tests. The odds ratio (OR) with a 95% confidence interval (CI) was taken as a measure of effect size. A 2-tailed p value of less than 0.05 was considered to indicate statistical significance. All statistical analyses (PCA) were performed using IBM SPSS statistics version 22. (SPSS Inc. Chicago, IL) and R version 3.2.3. In the case of microbiome analysis, alpha diversity was conducted using specaccum program in the vegan package^28^ as implemented for R version 3.2.3. R Packages ggplot2^29^, DESeq2^30^ and ggbiplot^31^ were used for the analysis. Diversity was analyzed by using QIIME microbiome pipeline^32^.

### Availability of data and material

The datasets supporting the conclusions of this article are included and available on line. Raw fastq data will be available upon request to the corresponding author.

## Electronic supplementary material

Supplementary Table 1

Supplementary Table2

Supplementary Table3

Supplementary Table4

Supplementary Table5

Supplementary Table7

Supplementary Table8

Supplementary Figure S1

Supplementary Figure S2
