# Supplementary material for: Gut microbial composition in patients with psoriasis
Source: Sci Rep. 2018 Feb 28;8:3812. doi: 10.1038/s41598-018-22125-y (PMC5830498; doi:10.1038/s41598-018-22125-y)
Supplement: Supplementary file 1 — Supplementary Table 1 [file 41598_2018_22125_MOESM1_ESM.docx]

**Gut microbial composition in patients with psoriasis**

Francisco M. Codoñer^1^, Ana Ramírez-Bosca^2,3*^, Eric Climent^1^, Miguel Carrión-Gutierrez^4^, Mariano Guerrero^2^, Jose Manuel Pérez-Orquín^5^, José Horga de la Parte^6^, Salvador Genovés^7^, Daniel Ramón^1,7^, Vicente Navarro-López^2,8*^ and Empar Chenoll^7*^

**Table 1. Baseline characteristics of patients with psoriasis**

| **Demographics** | **Study group patients (n=52)** |
| --- | --- |
| Age | 41.2 ± 14.4 |
| Age of diagnosis | 23.1 ± 13.9 |
| Gender (male) | 25 (48.1) |
| **Medical History** |  |
| Allergic rhinitis | 4 (7.7) |
| Dyspepsia and hiatal hernia | 3 (5.8) |
| Hypercholesterolemia | 3 (5.8) |
| High blood pressure | 3 (5.8) |
| Depression | 2 (3.8) |
| Thyroid disease | 2 (3.8) |
| Atopic dermatitis | 1 (1.9) |
| Hyperthyroidism | 1 (1.9) |
| β-lactam allergy | 1 (1.9) |
| **Analytical Characteristics** |  |
| Glucose (mg/dL) | 90.7 ± 19 |
| Serum Creatinine (mg/dL) | 0.9 ± 0.5 |
| Blood WBC/mm^3^ | 8700 ± 3402 |
| C-reactive protein test (mg/L) | 2.3 ± 0.3 |
| **Severity of Psoriasis** |  |
| (PASI score) | 13.3 ± 3.3 |

Definition of abbreviations: WBC, white blood cells; PASI, Psoriasis Severity Index. Data are expressed as mean ± SD and percentages (%) when applicable.
