# Supplementary figures and images for: Gut microbial composition in patients with psoriasis
Source: Sci Rep. 2018 Feb 28;8:3812. doi: 10.1038/s41598-018-22125-y (PMC5830498; doi:10.1038/s41598-018-22125-y)

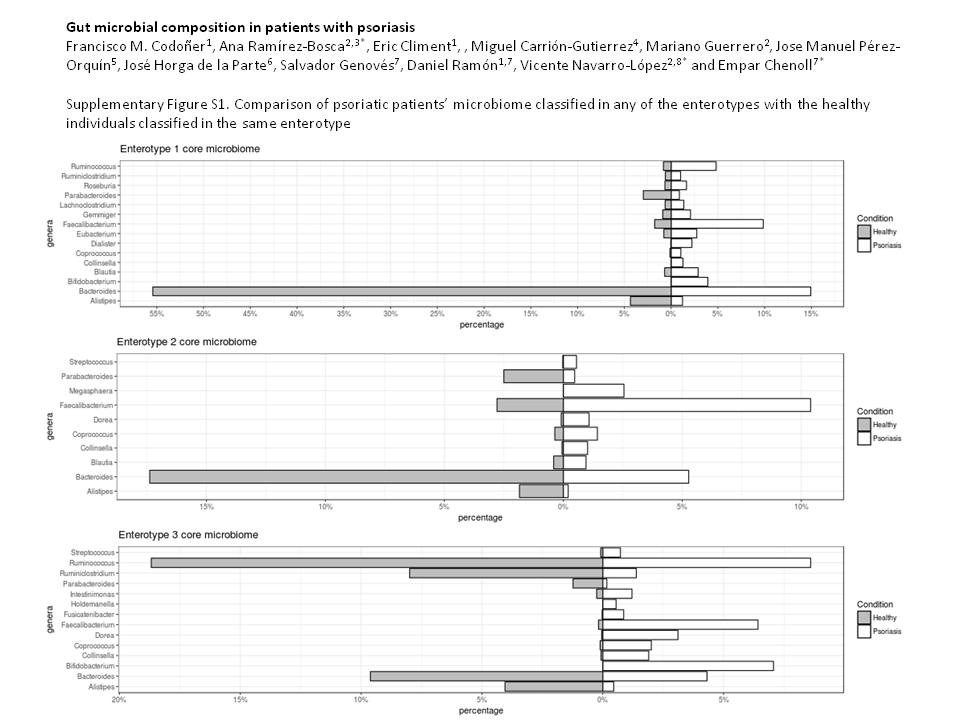

Supplement: Supplementary file 8 — Supplementary Figure S1 [file 41598_2018_22125_MOESM8_ESM.tif]

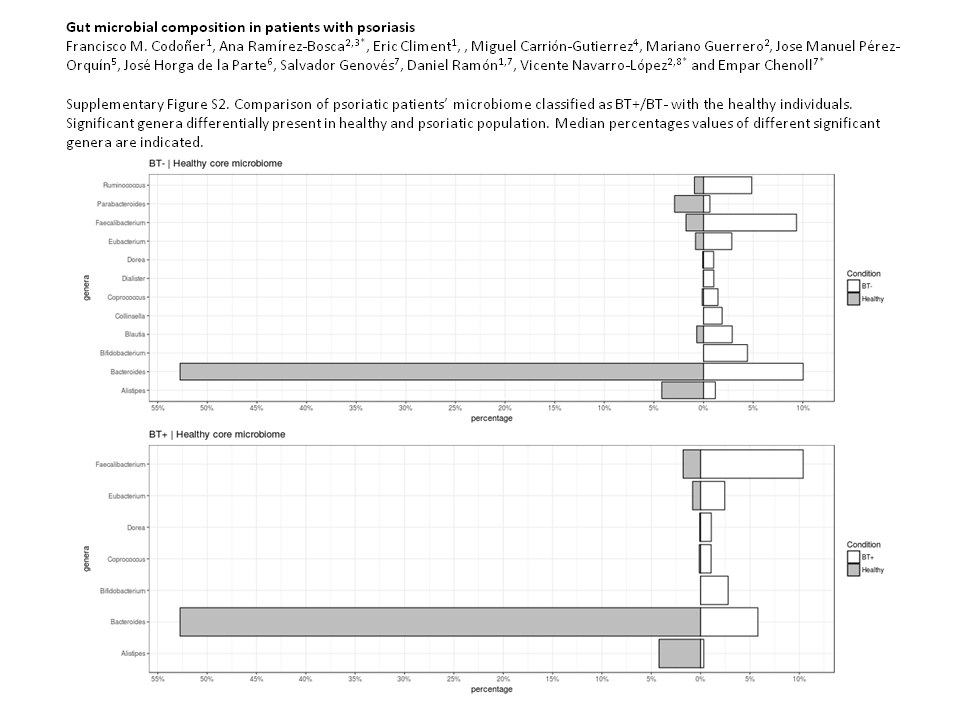

Supplement: Supplementary file 9 — Supplementary Figure S2 [file 41598_2018_22125_MOESM9_ESM.tif]
